# Meeting the Unmet Need in the Management of MDR Gram-Positive Infections with Oral Bactericidal Agent Levonadifloxacin

**DOI:** 10.1155/2022/2668199

**Published:** 2022-09-09

**Authors:** Yatin Mehta, K. C. Mishra, Yashesh Paliwal, Pradeep Rangappa, Sharmili Sinha, Sandeep Bhapkar

**Affiliations:** ^1^Medanta the Medicity, Gurgaon, India; ^2^Yashoda Hospital, Secunderabad, India; ^3^Fortis Hospital, Kolkata, India; ^4^Manipal Hospital, Bengaluru, India; ^5^Apollo Hospitals, Bhubaneshwar, India; ^6^Wockhardt Ltd, Mumbai, India

## Abstract

Levonadifloxacin (intravenous) and its oral prodrug alalevonadifloxacin are broad-spectrum antibacterial agents developed for the treatment of difficult-to-treat infections caused by multidrug-resistant Gram-positive bacteria, especially methicillin-resistant *Staphylococcus aureus*, atypical bacteria, anaerobic bacteria, and biodefence pathogens as well as Gram-negative bacteria. Levonadifloxacin has a well-defined mechanism of action involving a strong affinity for DNA gyrase as well as topoisomerase IV. Alalevonadifloxacin with widely differing solubility and oral bioavailability has pharmacokinetic profile identical to levonadifloxacin. Unlike existing MRSA drugs such as vancomycin and linezolid, which cause unfavorable side effects like nephrotoxicity, bone-marrow toxicity, and muscle toxicity, levonadifloxacin/alalevonadifloxacin has demonstrated superior safety and tolerability features with no serious adverse events. Levonadifloxacin/alalevonadifloxacin could be a useful weapon in the battle against infections caused by resistant microorganisms and could be a preferred antibiotic of choice for empirical therapy in the future.

## 1. Introduction

Diseases caused by multidrug-resistant (MDR) bacteria are associated with greater morbidity and mortality, and the use of current medications for previously treatable infections may become ineffective [1]. Methicillin-resistant *Staphylococcus aureus* (MRSA) is an important public health issue among the many resistant bacterial infections found throughout the world [2]. MRSA infections continue to be problematic in India, within both hospital and community settings [3]. Even though vancomycin, teicoplanin, and linezolid lack hallmarks of a “workhorse antibiotic” such as robust bactericidal action and a favorable safety profile, these remain the standard-of-care antibiotics for nosocomial MRSA infections. As a result, novel workhorse anti-MRSA treatments with an oral alternative for switch-over convenience are required.

There is also an unmet medical need in handling community MRSA infections. The management of community MRSA infections has further been hindered by the global introduction of a virulent, MDR, Panton-Valentine leucocidin-positive Bengal Bay clone (ST772-SCCmec type V; “SCCmec” stands for staphylococcal cassette chromosome mec) [4].

Levonadifloxacin (WCK 771), a benzoquinolizine fluoroquinolone, has a broad-spectrum activity against quinolone-resistant *Staphylococcus aureus* and MRSA phenotypes. In India, levonadifloxacin and its oral prodrug alalevonadifloxacin (WCK 2349) have recently been approved for the treatment of acute bacterial skin and soft structure infections (ABSSSI) with accompanying bacteremia and diabetic foot infections (DFI) [5]. These compounds have been subjected to several preclinical *in vitro* and *in vivo*, as well as clinical phase I studies to test their efficacy, safety, and toxicity. Several phase I trials have been performed in the United States (US) (ClinicalTrials.gov identifiers: NCT01875939, NCT02253342, NCT02244827, and NCT02217930), and a phase II study has been undertaken in India. In comparison to oral and intravenous linezolid, both oral and intravenous versions have been tested in India for the indication of ABSSSI and DFI (ClinicalTrials.gov identifier: NCT03405064).

This paper reviews the existing published data on levonadifloxacin and its prodrug alalevonadifloxacin, including relevant chemistry, mechanism of action, mechanism of resistance, microbiology, pharmacokinetics, pharmacodynamics, animal models, clinical trials, adverse effects, drug interactions, and their place in therapy. A comprehensive search of PubMed and Scopus was conducted using the search terms “levonadifloxacin and its prodrug alalevonadifloxacin” and “WCK 771” and “WCK 2349” to identify references for this review.

## 2. Multidrug Resistance and MDR Gram-Positive Infection

Antibiotics' efficacy, which has revolutionized medicine and saved millions of lives, is in jeopardy due to the increasing rise of resistant bacteria around the world. MDR has become a major issue in recent years, since the rate at which new antibiotics are developed has decreased dramatically while antibiotic use has increased [6, 7]. Each year, MDR infections kill at least 50,000 people in Europe and USA alone, with hundreds of thousands more dying in other parts around the globe. According to a UK Government-commissioned Review, it is anticipated that MDR might kill 10 million people each year by 2050, resulting in a total economic output of $100 trillion USD [8, 9].

The global spread of drug resistance among *Staphylococcus aureus* and *Enterococcus* species along with common respiratory pathogens like *Streptococcus pneumoniae* and *Mycobacterium tuberculosis* has reached epidemic proportions.

Many common antibiotics are becoming resistant to vancomycin-resistant enterococci (VRE) and a growing number of other pathogens. The majority of human enterococcal infections are caused by *E. faecalis* and *E. faecium*, which are also a primary cause of hospital-acquired and MDR infections. In patients with no risk factors, *E. faecalis* can cause community-acquired endocarditis [10]. A study on bacterial isolates obtained from patients of nursing facilities demonstrated 11.7% prevalence of *E. faecalis* colonization, acquired at a rate of 4.1 cases per 1000 person-days, with an inferred duration of carriage of 32 days [11]. Another study showed *E faecalis* prevalence of 8.6% and high rates of resistance to gentamicin, erythromycin, and vancomycin among Egyptian patients with hospital-acquired infections [12].

## 3. *Staphylococcus aureus*: A Gram-Positive Pathogen of Particular Concern

*Staphylococcus aureus* is a common cause of both hospital-acquired and community-acquired infections as well as skin and soft-tissue infections in both healthy people and those with risk factors or underlying conditions [13]. Due to its propensity to survive within monocytes and phagocytes, such as endothelial cells, epithelial cells, fibroblasts, osteoblasts, and keratinocytes, this pathogen has been proven to be the source of persistent infections at diverse anatomical sites [14]. MRSA can cause difficult-to-treat staph infections because of resistance to some antibiotics [15]. Over time, MRSA infections have become more common worldwide. MRSA isolates were first found in 1961 from United Kingdom [16]. The percentage of *S. aureus* infections worldwide ranges from 13 to 74%. In USA, the incidence rate of invasive MRSA infections in 2005 was found to be 31.8 per 100,000, where *S. aureus* bacteremia was the primary cause of 75% of these infections [17]. As per global surveillance report from the South-East Asia and Western Pacific Region, incidence rate of MRSA was found to be 2.3–69.1% [18, 19]. Due to an increase in community-acquired infections, the prevalence of MRSA bacteremia rose between 2000 and 2008 in Canada, Australia, and Scandinavia [18]. According to a recent systemic analysis, MRSA accounted for >100,000 deaths and 3.5 million disability-adjusted life years (DALYs) globally in 2019, associated with antimicrobial resistance [20]. A recent meta-analysis in India observed 37% total prevalence with pooled prevalence of MRSA varying between 31 and 39% during 2015–2019 and 69% in 2020 [21]. Another study showed a continuous rising trend of MRSA in different clinical samples from North India over 3 years (28% in 2017 to 35.1% in 2019) [22]. Several studies have found that patients infected with MRSA have a higher 30-day and 90-day mortality risk, as well as a 1.19-fold increase in hospital expenses when compared to those infected with methicillin-susceptible *S. aureus* (MSSA) [23, 24]. CDC and PHAC consider MRSA to be a serious threat and a high priority, respectively [13, 15].

MRSA strains were once restricted to hospitals, that is, hospital-associated MRSA (HA-MRSA); however, in the last 20 years, MRSA have emerged in the general populations (through variation and recombination) in diverse communities (community-associated MRSA or CA-MRSA) [25]. HA-MRSA and CA-MRSA differ not only in terms of clinical characteristics and molecular biology but also in terms of antibiotic susceptibility and treatment. Genotypically, CA-MRSA are newer and more virulent strains (with types IV or V SCC*mec* and Panton-Valentine leucocidin (PVL) encoding genes) and are generally susceptible to non-*β*-lactam antimicrobials [25–27].

MRSA's emergence is multifactorial, where host factors, infection control practices, and antimicrobial pressures play a major role. Bacterial mutation leading to the emergence of bacterial resistance phenotypes is associated with the clinical use of antimicrobial agents to which the bacteria are resistant. Bacteria that survive treatment with one antibiotic develop resistance to the effects of that drug and similar drugs [28, 29]. A study revealed that a change in normal colonizing flora (from MSSA to MRSA) of an individual occurs within 24–48 hours under selective antibiotic pressures [30]. Prolonged length of hospitalization, intensive care admission and recent or current hospitalization, recent or long-term antibiotic use, MRSA colonization, invasive procedures (such as urinary catheters, intra-arterial lines, or central venous lines), people with weak immune system (such as HIV infection), admission to nursing homes, open wounds, hemodialysis, and discharge with long-term central venous access or long-term indwelling urinary catheter are all risk factors for MRSA infection, contributing to an overall rise in medical costs, which can be catastrophic in a nation like India [28, 31]. MRSA infection is also more common among healthcare personnel who have direct contact with patients infected with these bacteria [29, 32].

## 4. Management of MDR Gram-Positive Infection: An Unmet Medical Need

### 4.1. Available Antimicrobial Agents

Over many decades, antimicrobial medicines have been the cornerstone of treatment for bacterial infections. Several drugs, including glycopeptides (e.g., vancomycin and teicoplanin), linezolid, tigecycline, and daptomycin, and even some beta-lactams, such as ceftaroline and ceftobiprole, continue to be active against MRSA [33]. For severe or life-threatening infections, glycopeptides and lipopeptides (vancomycin, teicoplanin, and daptomycin) were the recommended treatment, with linezolid serving as a unique alternative for oral down-step therapy despite the lack of robust safety and pharmacokinetic data and the unpredictable MRSA-strains' susceptibility profile against these [34]. Quinupristin/dalfopristin, a streptogramin antibiotic, and linezolid, an oxazolidinone, appear to be effective against vancomycin-resistant Gram-positive bacteria strains [35]. A number of newer antimicrobial agents including fluoroquinolone antibiotics were approved for the treatment of MRSA and other MDR Gram-positive pathogens.

### 4.2. Issue with Currently Available Agents

An ideal antibiotic should have a broad-spectrum bactericidal activity along with no teratogenic effects and drug-drug interactions. Despite missing hallmarks of a “workhorse antibiotic” such as robust bactericidal action and a favorable safety profile, vancomycin, daptomycin, teicoplanin, and linezolid have been considered to be the standard-of-care antibiotics against MRSA infections. However, vancomycin has significant drawbacks, such as relatively weak bactericidal activity, varying MICs, accompanying therapeutic failure, poor pharmacokinetic properties, and the risk for serious toxicity. Moreover, its usage has been restricted in recent years by the emergence of both tolerant and resistant species [36]. Furthermore, studies have shown association of vancomycin with production of hypersensitivity reactions, including anaphylaxis and “red man syndrome” and high-dose vancomycin therapy with the incidence of nephrotoxicity [36–38]. Vancomycin also shows poor penetration to certain body tissues, notably cerebrospinal fluid (CSF) [39]. Although antibacterial, daptomycin is ineffective in pneumonia and concomitant bacteremia [40]. Daptomycin has demonstrated comparable efficacy to vancomycin in complicated SSTIs, endocarditis, and MRSA bacteremia but not in pneumonia because of inactivation by alveolar surfactant [41]. However, reports have suggested rising daptomycin MICs of 1–2 *μ*g/mL in association with vancomycin-intermediate *Staphylococcus aureus* (VISA) and heteroresistant VISA (hVISA) strains, thereby raising concerns for cross resistance between daptomycin and vancomycin in hVISA and VISA [42]. Increased MIC of daptomycin was also found to be associated with increased mortality in patients with MRSA bacteremia. Last but not least, daptomycin has been linked to elevated creatine kinase levels and rhabdomyolysis [43], which is troublesome in critically ill patients who are already at risk for these increases and their side effects, like renal injury.

Though linezolid is easily administered orally, prolonged therapy is frequently linked with myelosuppression, necessitating blood parameter monitoring. Recent data imply that linezolid may be unsafe in patients with renal impairment due to its overexposure [44]. Furthermore, because of its weak bactericidal activity, linezolid is not recommended for immunosuppressed or bacteremia patients [44]. Clindamycin use for community MRSA infections is similarly limited due to high prevalence of inducible macrolide resistance in MRSA and numerous reports of antibiotic-associated colitis and antibiotic-associated diarrhea [44, 45].  Table 1 summarizes the common limitations of the currently available anti-MRSA agents.

### 4.3. Empirical MDR Gram-Positive Coverage

Empirical therapy with broad-spectrum antimicrobials plays a major role in the pharmacotherapy of complicated skin and soft-tissue infections (SSTIs), postsurgical site infections, and potential drug-resistant organisms like MRSA [46, 47]. Unlike definitive treatment, empirical or presumptive anti-infective therapy is one-time treatment administered for a presumed infection which is based on a clinical diagnosis along with evidence from the literature and educated experience with the bacteria that are likely to cause the infection [48]. When starting empiric antibiotics, it is critical that this therapy be started as soon as possible and as appropriately as possible, because delays in treatment are correlated with adverse outcomes [47, 48].

An initial empiric therapy utilizes broad-spectrum antimicrobial medicines in order to cover many potential infections linked with the specific clinical condition. However, once laboratory findings of microbiology tests with pathogen identification and antimicrobial susceptibility data are known, every effort should be undertaken to narrow the antibiotic spectrum. This is an important component of antimicrobial therapy, since it can lower cost and toxicity while also considerably delaying the emergence of antibiotic resistance in the community [48].

Thus, every effort should be made to carefully select antibiotics, balancing the necessity for wide empiric coverage of possible bacteria with the need to preserve existing antibiotics for when they are absolutely necessary. Current antibiotics' resistance or safety deficiency-related restrictions, as well as halted anti-infective drug research, raise the possibility of diverse resistance mechanisms spreading globally. Therefore, there is an unmet need for the development of novel treatments that are effective against a broad spectrum of multidrug-resistant Gram-positive bacteria.

## 5. Levonadifloxacin: New Agent for MDR Gram-Positive Pathogen (MRSA)

**Figure 1:**
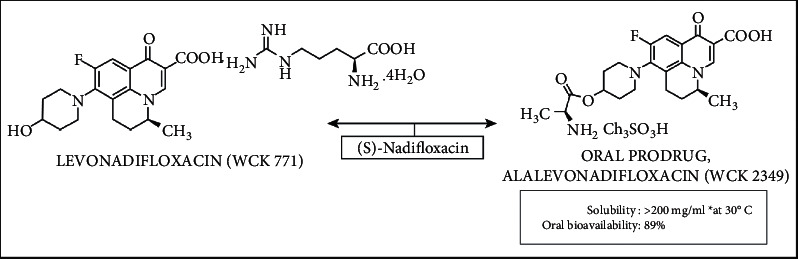
Structure of injectable and oral prodrug of levonadifloxacin.

After a 14-year hiatus, the two forms of novel anti-MRSA agent “levonadifloxacin,” intravenous and oral, have recently been launched in India by Wockhardt Limited as EMROK and EMROK O, respectively. The structure of injectable and oral prodrug of levonadifloxacin is shown in Figure 1.

Both forms have been recently approved by Drug Controller General of India and are licensed for the treatment of ABSSSI coexisting with bacteremia as well as DFI. The important features of levonadifloxacin are summarized in Table 2.

## 6. Levonadifloxacin: Mechanism of Action

**Figure 2:**
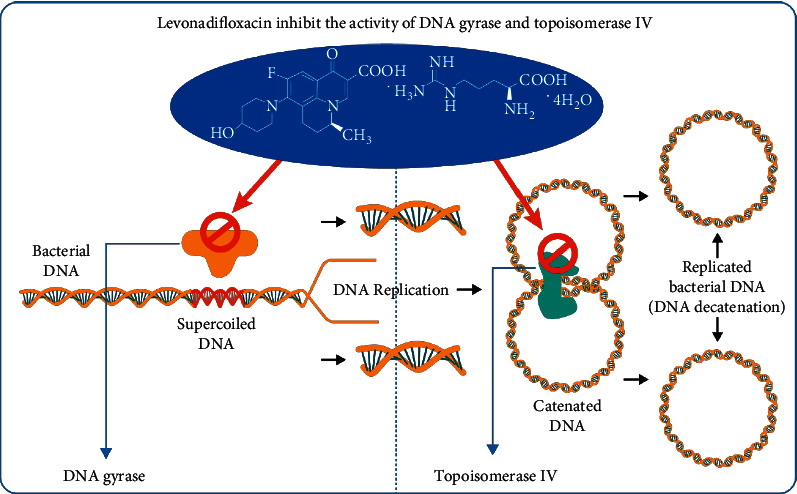
The mechanism of action of levonadifloxacin.

The Korean Society of Infectious Diseases and the Korean Society for Chemotherapy's 2018 guidelines highly support the use of respiratory fluoroquinolones as empirical therapy, with a very high level of evidence [49]. Quinolones are known to have cidal activity by increasing the concentration of DNA gyrase and topoisomerase IV enzyme-DNA cleavage complexes. Because bound quinolone physically prevents future ligation reactions, the cleavage caused by these complexes results in permanent chromosomal breakage. When a significant number of DNA strands break, other DNA repair processes are overwhelmed, resulting in bacterial cell death [50]. It is believed that, because of the presence of chiral benzoquinolizine core, levonadifloxacin shows a strong affinity for DNA gyrase, while retaining significant affinity towards topoisomerase IV. In contrast to other quinolones, such as ciprofloxacin and levofloxacin, which largely block DNA topoisomerase IV, levonadifloxacin has a well-defined mechanism of action involving a strong affinity for staphylococcal DNA gyrase as well as topoisomerase IV as shown in Figure 2 [51, 52].

## 7. Levonadifloxacin: Spectrum of Activity

Levonadifloxacin is a fluoroquinolone benzoquinolizine having broad-spectrum action against respiratory Gram-positive and Gram-negative pathogens, including methicillin- and quinolone-resistant *Staphylococcus aureus*, *Streptococcus pneumoniae*, *Streptococcus pyogenes*, *Haemophilus influenzae*, and *Moraxella catarrhalis*, and also against atypical pathogens such as *Mycoplasma pneumoniae, M. genitalium, M. hominis, Ureaplasma* spp. (including macrolide-, tetracycline-, and levofloxacin-resistant strains), *Chlamydophila pneumoniae*, and *Legionella pneumophila* (Table 3). A study demonstrated high activity against contemporary Gram-positive pathogens collected from various Indian hospitals [51]. It even has clinical benefits against quinolone-susceptible Gram-negative bacteria such as *E. coli*, *Klebsiella pneumoniae, Pseudomonas*, and *Acinetobacter* [50, 53]. Bioterror organisms such as *Bacillus anthracis, Francisella tularensis*, *Burkholderia mallei*, *Yersinia pestis*, and *Burkholderia pseudomallei* have also been shown to be susceptible to levonadifloxacin [44]. Levonadifloxacin has the benefit of being effective against resistant organisms with a very low mutation rate [54, 55]. Because of its large concentrations in the lungs and powerful intracellular activity against a wide range of possible respiratory pathogens, levonadifloxacin may be ideally suited for the treatment of extracellular and intracellular bacterial pathogen-caused respiratory infections, particularly in COVID-19 [56]. Levonadifloxacin not only kills biofilm-embedded QRSA and MRSA but also inhibits the NorA efflux pump, which is an important big contributor of development of quinolone resistance [57].

According to a study, WCK 771 exhibit bactericidal activity against vancomycin-resistant *S. aureus* strain HMC3 isolated from a patient's heel wound at the Hershey Medical Center, USA [58]. A study demonstrated the immunomodulatory effects of levonadifloxacin. In a lipopolysaccharide-stimulated human whole-blood (HWB) model, levonadifloxacin dramatically reduced inflammatory responses by inhibiting proinflammatory cytokines such as TNF-*α*, IL-6, and IL-1*β*, and, in mouse acute lung injury (ALI) model, it reduced lung total white blood cell count, myeloperoxidase, and cytokine levels, with peak effect observed mostly at 24 h [53]. The favorable safety and efficacy of levonadifloxacin in subjects receiving medications for comorbidities such as antidiabetics or antihypertensives show low drug-drug interaction, which is due to levonadifloxacin's lack of CYP interaction [59].

## 8. Meeting the Unmet Need with Levonadifloxacin IV and Oral

An oral version with equal PK/PD was required as a step-down therapy and therefore the L-arginine salt formulation (alalevonadifloxacin or WCK 2349) was created. It has a high oral bioavailability (89%) and has been successfully developed and launched as levonadifloxacin oral prodrug formulation (EMROK O) in India [60, 61]. Multiple phase I studies have been performed with levonadifloxacin IV and oral in India and the USA. A brief account of phase I studies is discussed in the following sections.

### 8.1. Phase I Indian Trial

A study was performed in India with a goal of investigating the pharmacokinetics of intravenous WCK 771. Healthy adult male subjects were administered single doses of ECK 771 ranging from 50 mg to 1200 mg, multiples doses of 500 mg and 600 mg twice daily for 1 day, and 600 mg to 1200 mg twice daily for 5 days. The results of this study showed that there was a linear increase in *C*_max_ and AUC_(0–∞)_ for 50 mg to 1200 mg single doses. *C*_max_ ranged between 1.84 and 32.33 *μ*g/mL and AUC_(0–∞)_ ranged between 10.07 and 277.66 *μ*g·hr/mL. The AUC_(tau)_ at steady state for BID for 5 days was not statistically different from the AUC_(0–∞)_ following corresponding single doses at all four dose levels. Accumulation could be seen after several doses. Furthermore, throughout single dose and multiple doses, the terminal elimination half-life (*t*_1/2_) remained constant at around 6–8 hours [62].

### 8.2. Phase I US Trial

Two separate trials in USA were conducted to evaluate the safety, pharmacokinetics, and tolerability of multiple ascending (twice daily at 12-hour intervals for 5 days) doses of WCK 771 (600, 800, or 1000 mg) and WCK 2349 (800, 1000, or 1200 mg). Mean total and peak exposures of levonadifloxacin increased from 800 to 1000 mg after WCK 2349 but the values remained relatively unchanged from 1000 to 1200 mg. The mean *t*_1/2_ of levonadifloxacin was comparable across dosages and ranged from 9.7 to 10.4 hours and from 8.28 to 9.62 hours for WCK 771 and WCK 2349, respectively. There were no deaths or serious adverse events in both studies. Therefore, both WCK 771 and WCK 2349 administered in multiple escalating doses were well tolerated by the US subjects [63]. The steady-state volume of distribution (V_ss_) ranged from 145.34 to 172.0 L after intravenous WCK 771 injection, the clearance (CL_ss_) ranged from 6.7 to 8.2 L/h, and the terminal *t*_1/2_ was 8.5–12 hours. The accumulation factor ranged from 0.99 to 1.1 over 5 days, indicating negligible or minimal buildup [64].

### 8.3. Phase I Study: Intrapulmonary Pharmacokinetics of Levonadifloxacin

A phase I study was conducted in healthy adult human subjects with an aim to compare plasma, epithelial lining fluid (ELF), and alveolar macrophage (AM) concentrations of levonadifloxacin following oral administration of alalevonadifloxacin (1000 mg twice daily for 5 days). The penetration ratios for ELF and AM to plasma concentration for levonadifloxacin were 7.66 and 1.58, respectively, supporting its use for lower respiratory tract infections. Oral levonadifloxacin's elimination half-life, clearance, and volume of distribution were found to be 6.35 h, 8.17 L/hour, and 59.2 L, respectively. The phase I study concluded that the oral administration of alalevonadifloxacin at a dose of 1,000 mg twice day for 5 days was shown to be safe and well tolerated (NCT02253342) [61].

### 8.4. Phase I Study: Drug-Food Interaction

Furthermore, Bhagwat et al. investigated the influence of food on WCK 2349 oral absorption and assessed the absolute bioavailability of WCK 2349 at 1000 mg in comparison to 800 mg WCK 771 administered as an intravenous infusion. WCK 2349 given in the fed condition reduced *C*_max_ by 27% compared to the fasted state and delayed the time to achieve *C*_max_ (*T*_max_) by 2 hours. The AUC values, on the other hand, remained unchanged. The study demonstrated that WCK 2349 could be delivered regardless of fed or fasted state. WCK 2349 had an absolute bioavailability of 89.35 percent and similar concentration-time profile of levonadifloxacin when compared to WCK 771. In a nutshell, these findings suggest that switching from an intravenous to an oral formulation of levonadifloxacin is effective for inpatients (NCT01875939) [50, 65].

### 8.5. Phase I Study: Effect on QT Interval

In another study, the electrocardiographic (ECG) effects of WCK 2349 at a supratherapeutic oral dose of 2,600 mg in 48 normal participants were compared to placebo and oral moxifloxacin (400 mg). WCK 2349 had no effect on baseline and placebo-corrected QTcF (QT interval adjusted for heart rate using the Fridericia method), QRS, or PR interval. Except for a possibly transient elevation in HR, which appears to be clinically negligible, a supratherapeutic dose of WCK 2349 is not likely to elicit clinically significant ECG effects and thus can provide a viable alternative to QT prolonging antibiotics (NCT02217930) [66].

### 8.6. Phase I Study: PK in Hepatic Impairment

Another phase I trial was conducted to understand the pharmacokinetics of levonadifloxacin and alalevonadifloxacin in patients with hepatic impairment. The study data suggested that WCK 771 and WCK 2349 could be safely administered to patients with hepatic impairment in order to obtain a therapeutically suitable PK profile (NCT02244827) [50].

## 9. Potential Benefits of Levonadifloxacin

In nonclinical and clinical investigations, levonadifloxacin and its prodrug alalevonadifloxacin (WCK 2349, oral) have been studied for the treatment of ABSSSI, CABP, and other types of infections.

### 9.1. In ABSSSI with DFI

A multicentric phase 3 trial which was an active-comparator study was completed recently. The aim of the study was to establish the noninferiority of oral levonadifloxacin (1000 mg) with oral linezolid (600 mg) and the noninferiority of IV levonadifloxacin (800 mg) with IV linezolid (600 mg) in ABSSSI, including diabetic foot infection at Test of Cure (TOC) visit. Before the commencement of this phase 3 trial, 157 subjects (healthy volunteers) in multiple phase I studies (115 subjects from India and 42 subjects from USA) and 104 subjects in phase II (India) studies received IV levonadifloxacin, and 287 subjects (263 healthy volunteers and 24 hepatically impaired subjects) in multiple phase I studies (94 subjects from India and 193 subjects from USA) and 119 subjects in phase II (India) studies received oral levonadifloxacin.

When compared to linezolid (IV and oral), levonadifloxacin (IV and oral) exhibited a greater clinical responder rate of 85.2% and 92.7% during visit 3 (days 3-4). The clinical cure rate at TOC was also found to be higher in levonadifloxacin (IV and oral) when compared to linezolid (IV and oral), that is, 95% versus 89.3%, respectively, for MRSA patients, indicating its favorable microbiological efficacy. Additionally, in the diabetic foot ulcer subgroup, clinical cure at TOC for levonadifloxacin IV was higher than that for linezolid (91.7% versus 76.9%). The pharmacokinetic investigation revealed that the bioavailability of oral levonadifloxacin was 90%, and the comparable pharmacokinetic profile of levonadifloxacin by both routes provides an alternative for switch from IV to oral treatment. Mild constipation (3.6%), hyperglycemia (1.6%) of mild-to-moderate severity, and being not related to levonadifloxacin as majority of these patients had high blood glucose at screening and cough (1.2%) (mild severity and not related to levonadifloxacin) were the most common AEs reported in levonadifloxacin-treated subjects. Overall, both IV and oral levonadifloxacin treatments were well tolerated and noninferior to IV and oral linezolid in participants with ABSSSI (NCT03405064; CTRI No.: CTRI/2017/06/008843) [67]. The PIONEER study also indicated remarkable clinical success rates of 98.2% for ABSSSI and 95.1% for DFI [68].

### 9.2. In CABP/Lower Respiratory Tract Infection

A multicenter, retrospective, postmarketing, real-world study as a part of PIONEER study was conducted to document the outcomes of oral and/or intravenous administration of levonadifloxacin for the treatment of lower respiratory tract infection (LRTI), in particular CABP in hospital and outpatient settings. 338 pneumonia/LRTI patients included in the study were given levonadifloxacin as empirical therapy with clinical success rates for the drug as 93.5% with intravenous therapy, 98.7% with oral therapy, and 100.0% with intravenous followed by oral therapy. Moreover, investigators graded levonadifloxacin therapy as “excellent to good” for efficacy in 95.2% of patients and “very good” for safety in 97.9% of patients. Only 4 (1.2 percent) of the 338 individuals who received levonadifloxacin had a total of 5 (1.5 percent) minor adverse events, with nausea in three patients and diarrhea and fatigue in one patient each. The study thus displayed a favorable PK/PD and safety profile of levonadifloxacin in the case of LRTI/CABP [59].

### 9.3. In Febrile Neutropenia

Years of empirical antibiotic treatment have resulted in a shift in infection pathogens from primarily Gram-negative bacteria to more Gram-positive bacteria. Empirical antibiotic therapy in the treatment of fever and neutropenia reduces the risk of developing sepsis, septic shock, acute respiratory distress syndrome, organ failure, and mortality [69]. Fluoroquinolone antimicrobial prophylaxis has been shown to reduce the incidence of neutropenic fever, infection rates, hospitalization rates, and length of hospital stay in this patient population [70]. Fluoroquinolone prophylaxis also lowers febrile neutropenia in patients with solid tumors or lymphoma receiving cyclical standard-dose myelosuppressive chemotherapy [71]. Levonadifloxacin has also proved to be an effective agent for prophylaxis of febrile neutropenia because of its good safety and efficacy profile. The clinical success rate for treating febrile neutropenia with levonadifloxacin medication (oral/IV) was found to be 93.8% in the PIONEER research [72].

### 9.4. In Bone and Joint Infections

*Staphylococcus aureus* is the primary cause of bone and joint infections (BJI). BJI caused by MRSA necessitates the use of antibiotics for a longer period of time, which cannot be done safely with the present anti-MRSA drugs due to their adverse events. Studies have shown excellent levonadifloxacin's resistance mechanisms such as the NorA efflux pump, DNA Gyrase/Topo IV mutations, and biofilm formation, which indicate that this drug can potentially be used to treat difficult-to-treat BJI [68, 73]. Levonadifloxacin demonstrated very good results in PIONEER study for BJI, with clinical success rates of 100% [72]. Additional research works employing BJI animal models as well as human trials are required to thoroughly examine levonadifloxacin therapy and its positioning in the treatment of these infections. The various clinical trials of levonadifloxacin and alalevonadifloxacin are summarized in Table 4.

## 10. Superiority of Levonadifloxacin over Other Agents

Unlike other fluoroquinolones (moxifloxacin, levofloxacin, and ciprofloxacin) that degrade in acidic environments, levonadifloxacin showed improved activity at pH 5.5, increasing its therapeutic potential in intracellular infections and other clinical situations with acidic environments. The MIC of levonadifloxacin against *S. aureus* strains was 2, 8, and 16 times lower than those of moxifloxacin, levofloxacin, and ciprofloxacin, respectively, at pH 7.4 which was further reduced at pH 5.5. On the contrary, comparator quinolones saw a fourfold increase in MIC at pH 5.5 [14].

Levonadifloxacin had a steady bacterial death rate of 90% against methicillin- and quinolone-resistant *Staphylococcus aureus* embedded biofilms; clindamycin and linezolid had inconsistent efficacy, whereas vancomycin and daptomycin had no activity. Scanning electron microscopy images verified levonadifloxacin's efficiency against biofilm, demonstrating disruption of biofilm structure and a concomitant reduction in viable bacterial population [74].

In another study, WCK 771 showed the NorA efflux pump had no effect on the activity of WCK 771, indicating it as a major advantage, since a high number of *staphylococcal* isolates exhibit efflux-mediated fluoroquinolone resistance [75].  Table 5 compares the efficacy and side effects of currently available anti-MRSA drugs, including levonadifloxacin, vancomycin, teicoplanin, linezolid, daptomycin, ceftaroline, and omadacycline.

## 11. Conclusion

Levonadifloxacin (intravenous) and oral prodrug of levonadifloxacin, that is, alalevonadifloxacin, are broad-spectrum anti-MRSA benzoquinolizine subclass of quinolones. Both IV and oral forms have been studied for the treatment of ABSSSI, CABP, and other types of infections and phase II and phase III clinical studies have been completed, demonstrating that they are clinically acceptable therapeutic options for the management of complex and serious infections caused by MDR Gram-positive bacteria, especially MRSA with a very low frequency of mutation, atypical bacteria, anaerobic bacteria, and biodefence pathogens, as well as Gram-negative bacteria. Alalevonadifloxacin is formulated to release the active drug immediately after oral administration that is responsible for excellent uptake and bioavailability in both the fasting and fed states. The pharmacokinetic and pharmacodynamic properties of alalevonadifloxacin including improved aqueous solubility can help for an easy transition from parenteral to oral medication.

The existing MRSA drugs such as vancomycin, teicoplanin, daptomycin, and linezolid have unfavorable features such as nephrotoxicity, bone-marrow depression, and muscle toxicity and thus cannot be given to patients with compromised kidney/liver function or critically ill patients who require chronic therapy. However, unlike other MRSA drugs, clinical and nonclinical studies have established superior safety and tolerability features of levonadifloxacin/alalevonadifloxacin with no serious adverse events. Novel anti-MRSA agent like levonadifloxacin/alalevonadifloxacin is a therapeutic candidate for the management and treatment of difficult-to-treat infections caused by resistant pathogens and could be a preferred antibiotic of choice for empirical therapy. However, large-scale trials in India are needed to prove the efficacy and safety of levonadifloxacin therapy.

## Figures and Tables

**Table 1:** Common limitations of the currently available anti-MRSA agents.

Antibiotic	Mechanism of action	Limitations
Vancomycin	(i) Inhibits cell wall (peptidoglycan) synthesis (ii) Bactericidal activity (variable)	(i) MIC creep, hVISA development (ii) Variable tissue penetration (iii) Potential for nephrotoxicity at higher concentrations and in combination with other nephrotoxic agents (iv) Need for TDM

Daptomycin	(i) Disrupts cell membrane potential through rapid depolarization (ii) Bactericidal activity	(i) Inactivated by pulmonary surfactant, not effective treatment of MRSA pneumonia (ii) Potential for decreased susceptibility with increased vancomycin MIC and hVISA

Linezolid	(i) Inhibits protein synthesis through binding of 50S ribosomal subunit (ii) Bacteriostatic activity	(i) Multiple potentially serious side effects (marrow suppression, lactic acidosis, peripheral and optic neuropathy, serotonin syndrome), especially with prolonged use

Trimethoprim/sulfamethoxazole	(i) Inhibits multiple stages in bacterial folate and thymidine synthesis (ii) Bactericidal activity	(i) May be ineffective in infections involving undrained pus due to thymidine scavenging (ii) Limited data supporting use in bacteremia and endocarditis

Clindamycin	(i) Inhibits protein synthesis through binding of 50S ribosomal subunit (ii) Bacteriostatic activity	(i) Largely unproven for treatment of invasive infections in adults (ii) Inducible resistance can be missed if D-testing is not performed on clinical isolates (iii) Association with antibiotic-associated diarrhea and *Clostridium difficile* colitis

Tetracyclines	(i) Inhibit protein synthesis through binding of 30S ribosomal subunit (ii) Bacteriostatic activity	(i) Unproven for treatment of invasive infections

Tigecycline	(i) Inhibits protein synthesis through binding of 30S ribosomal subunit (ii) Bacteriostatic activity	(i) Low serum levels (ii) Probably not effective in treatment of HA-MRSA pneumonia

Quinupristin/dalfopristin	(i) Synergistic combination of two streptogramin compounds that inhibit protein synthesis (ii) Bactericidal activity in the absence of MLS_B_ resistance	(i) Frequent side effects (arthralgias, myalgias, venous intolerance) (ii) Multiple drug-drug interactions (iii) Limited data supporting use in invasive disease

Rifampicin	(i) Inhibits bacterial transcription (ii) Bactericidal activity	(i) Rapid development of resistance; cannot be used as monotherapy (ii) Multiple drug-drug interactions (iii) Potential hepatotoxicity

Teicoplanin	(i) Inhibits cell wall synthesis	(i) Nephrotoxicity (ii) MIC creep (iii) 2-3 days required to reach therapeutic levels, even with loading dose (iv) Variable tissue penetration (v) Dose adjustment required in renal patients (vi) TDM is recommended

Ceftaroline	(i) Binds to penicillin binding protein (PBP2a) and inhibits the synthesis of the peptidoglycan layer of bacterial cell walls	(i) Poor intracellular concentration (ii) Dose adjustment in renal patients (iii) Cannot be used as monotherapy in CABP (iv) *Clostridium difficile*-associated diarrhea

CABP: community-acquired bacterial pneumonia; HA-MRSA: hospital-associated MRSA; hVISA: heteroresistant vancomycin-intermediate *Staphylococcus aureus*; MIC: minimum inhibitory concentration; MLS_B_: macrolide-lincosamide-streptogramin B; MRSA: methicillin-resistant *Staphylococcus aureus*; TDM: therapeutic drug monitoring.

**Table 2:** Features of levonadifloxacin.

Antibiotic class	Benzoquinolizine fluoroquinolone
Administration route	Intravenous and oral
Intravenous dose regimen	800 mg BID
Oral dose regimen	1000 mg BID
Indications	ABSSSI with concurrent bacteremia and DFI
Activity spectrum	MRSA, QRSA, VRSA, VISA, Quinolone-S Gram-negatives, RTI pathogens
MRSA coverage	>99 percent
Gram-negative coverage	Partial
Intracellular activity	Yes (including MRSA + QRSA)
Biofilm eradication	Strong action for MRSA/QRSA biofilms
Activity in acidic conditions	Enhanced
*T *_max_	2.68 ± 1.27 h for oral 1000 mg
*C *_max_	21.48 ± 8.82 *μ*g/mL for oral 1000 mg
Plasma AUC	Highest plasma exposures among quinolones
	Intravenous 800 mg: 377.8 ± 35.33 mg·h/L
	Oral 1000 mg: 318.4 ± 33.2
Epithelial lining fluid AUC	Highest lung penetration among quinolones 1000 mg oral OD: 345.2 *μ*g·h/mL
Mean elimination half-life of 800 mg BID infused over 90 minutes	6.8 hours
Metabolism	72% of intravenous levonadifloxacin excreted as levonadifloxacin sulfate metabolite (approximately 50.3% in urine and 21.6% in faeces)
Dose adjustment in renal impaired patients	Not required^*∗*^
Dose adjustment in hepatic impairment	Not required
Liver safety	Very good
Cardiovascular system safety	Excellent
Gastrointestinal tolerability	Excellent

^*∗*^<5% of dose is excreted as unchanged levonadifloxacin suggesting minimal role of renal system in elimination of levonadifloxacin. ABSSSI: acute bacterial skin and skin structure infections; AUC: area under curve; BID: bis in die/twice a day; CAP: community-acquired pneumonia; cIAI: complicated intra-abdominal infections; *C*_max_: maximum mean plasma concentration; DFI: diabetic foot infections; MDR: multidrug-resistant; MRSA: methicillin-resistant *Staphylococcus aureus*; MSSA: methicillin-susceptible *Staphylococcus aureus*; QRSA: quinolone-resistant *Staphylococcus aureus*; RTI: respiratory tract infections; *T*_max_: time to reach maximum concentration; VISA: vancomycin-intermediate *Staphylococcus aureus*; VRSA: vancomycin-resistant *Staphylococcus aureus*.

**Table 3:** Unique multispectrum coverage of levonadifloxacin.

Spectrum of activity of levonadifloxacin	Organisms
Excellent Gram-positive bacteria coverage	(i) *Staphylococcus aureus*: MSSA, MRSA, QRSA, VRSA
	(ii) Coagulase-negative staphylococci
	(iii) *Staphylococcus epidermidis*
	(iv) *Streptococcus pneumoniae*
	(v) *Streptococcus pyogenes*
	(vi) *Streptococcus agalactiae*
	(vii) Viridans group *streptococci*

Quinolone sensitive Gram-negative (at par with ciprofloxacin)	(i) *Escherichia coli*
	(ii) *Klebsiella* spp.
	(iii) *Enterobacter* spp.
	(iv) *Citrobacter* spp.
	(v) *Proteus* spp.
	vi) *Providencia* spp.
	(vii) *Pseudomonas aeruginosa*
	(viii) *Moraxella catarrhalis*
	(ix) *Haemophilus influenzae*

Good anaerobic coverage	(i) *Clostridium difficile*
	(ii) *Clostridium perfringens*
	(iii) *Cutibacterium* (*Propionibacterium*) *acnes*
	(iv) *Peptostreptococcus* spp.

Good atypical bacteria coverage	(i) *Mycoplasma*
	(ii) *Chlamydia pneumoniae*
	(iii) *Chlamydia trachomatis*
	(iv) *Legionella pneumophila*
	(v) *Ureaplasma*

MRSA: methicillin-resistant *Staphylococcus aureus*; MSSA: methicillin-susceptible *Staphylococcus aureus*; QRSA: quinolone-resistant *Staphylococcus aureus*; VRSA: vancomycin-resistant *Staphylococcus aureus*.

**Table 4:** Summary of clinical studies with levonadifloxacin and alalevonadifloxacin.

Aim of the Study	Dose	No. of subjects	Study outcome
Crossover food-effect and absolute bioavailability study of alalevonadifloxacin [65]	800 mg (IV) and 1000 mg (oral)	12	Alalevonadifloxacin could be delivered regardless of fed or fasted state. Alalevonadifloxacin had concentration-time profile similar to that of levonadifloxacin. These findings suggest that switching from an intravenous to an oral formulation of levonadifloxacin is effective for inpatients

Determine the supratherapeutic dose of alalevonadifloxacin and assess its effect on cardiac repolarization as shown by analysis of the QT interval [66]	Part 1: Single dose of 1800 mg, 2200 mg, 2600 mg, and 3000 mg Part 2: Single dose of 2600 mg, moxifloxacin 400 mg, and placebo matched to moxifloxacin	Part 1: 32 (24 alalevonadifloxacin + 8 placebo) Part 2: 48	Supratherapeutic dose of alalevonadifloxacin is not likely to elicit clinically significant ECG effects and thus can provide a viable alternative to QT prolonging antibiotics

Evaluate the effect of hepatic impairment on the pharmacokinetics of levonadifloxacin and its sulfate metabolite after single oral dose administration of alalevonadifloxacin [50]	1000 mg	48	There were no significant differences (*p* > 0.05) in the PK parameters of levonadifloxacin or its sulfate metabolite in mild or moderate hepatic impaired groups compared to normal matched control groups. Levonadifloxacin and alalevonadifloxacin could be safely administered to patients with hepatic impairment in order to obtain a therapeutically suitable PK profile

Determine and compare plasma, ELF, and AM concentrations of levonadifloxacin after oral administration of alalevonadifloxacin [61]	1000 mg BID *x* 5 days	31	The penetration ratios for ELF and AM to plasma concentration for levonadifloxacin were 7.66 and 1.58, respectively, supporting its use for lower respiratory tract infections

Comparative study of levonadifloxacin (IV and oral) with linezolid (IV and oral) in ABSSSI [67]	Experimental: oral levonadifloxacin (1000 mg BID) or IV levonadifloxacin (800 mg BID) Active comparator: oral linezolid (600 mg BID) or IV linezolid (600 mg BID)	501	When compared to linezolid (IV and oral), levonadifloxacin (IV and oral) exhibited a greater clinical responder rate of 85.2% and 92.7%. The clinical cure rate was also found to be higher in levonadifloxacin (IV and oral) when compared to linezolid (IV and oral), i.e., 95% versus 89.3%, respectively, for MRSA patients, indicating its favorable microbiological efficacy. Additionally, in the diabetic foot ulcer subgroup, clinical cure for IV levonadifloxacin was higher than that for linezolid (91.7% versus 76.9%).

ABSSSI: acute bacterial skin and skin structure infections; AM: alveolar macrophage; BID: bis in die/twice a day; ECG: electrocardiogram; ELF: epithelial lining fluid; IV: intravenous; MRSA: methicillin-resistant *Staphylococcus aureus*; PK: pharmacokinetic.

**Table 5:** Comparative efficacy and safety parameters between anti-MRSA agents (Reddy et al. [76].

	Levonadifloxacin	Vancomycin/teicoplanin	Linezolid	Daptomycin	Ceftaroline	Omadacycline
Dose	800 mg BID (IV); 1000 mg BID (oral)	Vancomycin: 0.5 g QD or 1 g BID Teicoplanin: 400 mg BID (LD); 400 mg OD (MD)	600 mg BID	500 mg OD	600 mg BID	CAP: day 1: LD of 200 mg IV QD or 100 mg IV BID; day 2: MD of 100 mg IV QD or 300 mg PO QD SSTI: day 1: LD of 200 mg IV or 100 mg IV BID; day 2: MD of 100 mg IV QD or 300 mg PO QD OR SSTI (only for tablets): days 1 and 2: LD of 450 mg PO QD; day 3: MD of 300 mg PO QD

Spectrum	Broad	Narrow	Narrow	Narrow	Broad	Broad

Formulation	IV and oral	IV only	IV and oral	IV only	IV only	IV and oral

Bacterial killing	Cidal	Slow bactericidal	Static	Cidal	Cidal	Static

Major adverse effects	None	Nephrotoxicity	Bone-marrow suppression	Muscle toxicity	Diarrhea, nausea, and rash	Nausea, vomiting, infusion site reactions, alanine aminotransferase increased, aspartate aminotransferase increased, gamma-glutamyl transferase increased, hypertension, headache, diarrhea, insomnia, and constipation

Lung tissue concentration	Excellent	Poor	Good	Not active	Poor	Poor

MRSA Biofilm action	Yes	No	Moderate	No	No	No

Dose adjustment in RI	No	Yes	No	Yes	Yes	No

Dose adjustment in HI	No	No	Yes	No	No	No

BID: bis in die/twice a day; CAP: community-acquired pneumonia; HI: hepatic impairment; IV: intravenous; LD: loading dose; MD: maintaining dose; MRSA: methicillin-resistant *Staphylococcus aureus*; OD: once daily; PO: per os; QD: quaque die/once daily; RI: renal impairment.
